# Synthesis and mechanism-of-action of a novel synthetic antibiotic based on a dendritic system with bow-tie topology

**DOI:** 10.3389/fmicb.2022.912536

**Published:** 2022-08-26

**Authors:** Ainhoa Revilla-Guarinos, Philipp F. Popp, Franziska Dürr, Tania Lozano-Cruz, Johanna Hartig, Francisco Javier de la Mata, Rafael Gómez, Thorsten Mascher

**Affiliations:** ^1^Department of General Microbiology, Institut Für Mikrobiologie, Technische Universität Dresden, Dresden, Germany; ^2^Department of Organic and Inorganic Chemistry, Research Institute in Chemistry “Andrés M. Del Río” (IQAR), University de Alcalá, Madrid, Spain; ^3^Ramón y Cajal Health Research Institute (IRYCIS), Madrid, Spain; ^4^Networking Research Center on Bioengineering, Biomaterials and Nanomedicine (CIBER-BBN), Madrid, Spain

**Keywords:** drug design, mode of action, cell envelope stress response, carbosilane dendritic system, *Bacillus subtilis*, antimicrobial resistance, whole-cell biosensors

## Abstract

Over the course of the last decades, the continuous exposure of bacteria to antibiotics—at least in parts due to misprescription, misuse, and misdosing—has led to the widespread development of antimicrobial resistances. This development poses a threat to the available medication in losing their effectiveness in treating bacterial infections. On the drug development side, only minor advances have been made to bring forward novel therapeutics. In addition to increasing the efforts and approaches of tapping the natural sources of new antibiotics, synthetic approaches to developing novel antimicrobials are being pursued. In this study, BDTL049 was rationally designed using knowledge based on the properties of natural antibiotics. BDTL049 is a carbosilane dendritic system with bow-tie type topology, which has antimicrobial activity at concentrations comparable to clinically established natural antibiotics. In this report, we describe its mechanism of action on the Gram-positive model organism *Bacillus subtilis*. Exposure to BDTL049 resulted in a complex transcriptional response, which pointed toward disturbance of the cell envelope homeostasis accompanied by disruption of other central cellular processes of bacterial metabolism as the primary targets of BDTL049 treatment. By applying a combination of whole-cell biosensors, molecular staining, and voltage sensitive dyes, we demonstrate that the mode of action of BDTL049 comprises membrane depolarization concomitant with pore formation. As a result, this new molecule kills Gram-positive bacteria within minutes. Since BDTL049 attacks bacterial cells at different targets simultaneously, this might decrease the chances for the development of bacterial resistances, thereby making it a promising candidate for a future antimicrobial agent.

## Introduction

Bacteria are highly adaptable to changing environments, including exposure to antimicrobial agents. In modern medicine, the widespread misuse and overuse of known antibiotics increasingly gave rise to the spread of multidrug resistance bacteria. These “superbugs” threaten human health at accelerating pace. To counteract this development, the Global Action Plan on Antimicrobial Resistance developed by the World Health Organization has recently highlighted different strategic objectives to ensure antibiotic efficacy (WHO, 2015). Next to raising the awareness on the importance of administering correct dosing and duration of antibiotic treatments, to preserve their effectiveness, boosting research and development of new antimicrobial agents has been highlighted as a top priority goal of this campaign.

Regarding the latter objective, different experimental approaches can be undertaken. One strategy comprises further exploration and modification of compounds present in nature. Along these lines, antimicrobial peptides are currently regarded as promising resources to substitute classical antibiotics especially in the light of reported low rates of resistance developments (Draper et al., 2015; Ageitos et al., 2016; Jaumaux et al., 2020). Another promising strategy is to expand the access to the wealth of secondary metabolites produced, e.g., by Streptomycetes by a combination of genome mining and subsequent examination of the hidden antimicrobial potential of silent antibiotic clusters (Baltz, 2008). Modifying known chemical structures of commonly used antibiotics is yet another approach to increase the potency of such antimicrobial compounds relative to their natural antibiotic precursor (Brötz-Oesterhelt and Brunner, 2008). Special emphasis is laid on enhancing chemical properties, such as their stability, solubility, or lowering the toxicity (Power et al., 2008; Tevyashova et al., 2013). New formulations and approaches for drug delivery, such as lipid-association or encapsulation in polymeric nanoparticles, have the potential to reduce the dose-dependent toxic side effects of some natural antibiotics that lack clinical alternatives, thus extending their applicability in day-to-day routine medicine (Hamill, 2013; Palma et al., 2018).

Another strategy to tackle the antibiotic crises aims at collecting all current knowledge on natural antibiotics, their mode of actions and resulting bacterial mechanisms of resistances. This comprehensive knowledge-based approach then provides a framework for the rational *de novo* design and subsequent *in vitro* chemical synthesis of novel molecules fulfilling critical aspects that natural compounds lack (Fjell et al., 2012; Torres et al., 2019). Inevitably, the antimicrobial properties of these rationally designed synthetic antibiotics must be subsequently tested experimentally. Along those lines, an interesting group of molecules for developing *de novo* synthetic antimicrobials are carbosilane dendrimers.

Carbosilane dendrimers are well-defined single molecular weight structures (monodisperse) with carbon and silicon atoms-based scaffold and numerous terminal groups (multivalency) suited to engage in multivalent interactions that have a potential in multiple applications. That is, dendrimers are usually produced in an iterative sequence of reaction steps in which every additional iteration (generation) leads to a higher generation system that results in an exponential increase in molecular weights and peripheral functional groups. This type of dendritic structure shows a versatile and lipophilic skeleton able to load functional groups of different nature and interact with biological membranes by simple hydrophobic interactions. The biological effect of dendritic systems has been assayed based on their peripheral characteristics. Specifically, varied positively charged carbosilane dendrimers containing ammonium, guanidine, or biguanide moieties have shown good profile as antibacterial, antifungal, antiamoebic, or even antiamyloid systems. The systems act through an unspecific mechanism, for which the hydrophilic-hydrophobic balance is a parameter to be taken into account (Heredero-Bermejo et al., 2018; Fernandez et al., 2019).

**Figure 1:**
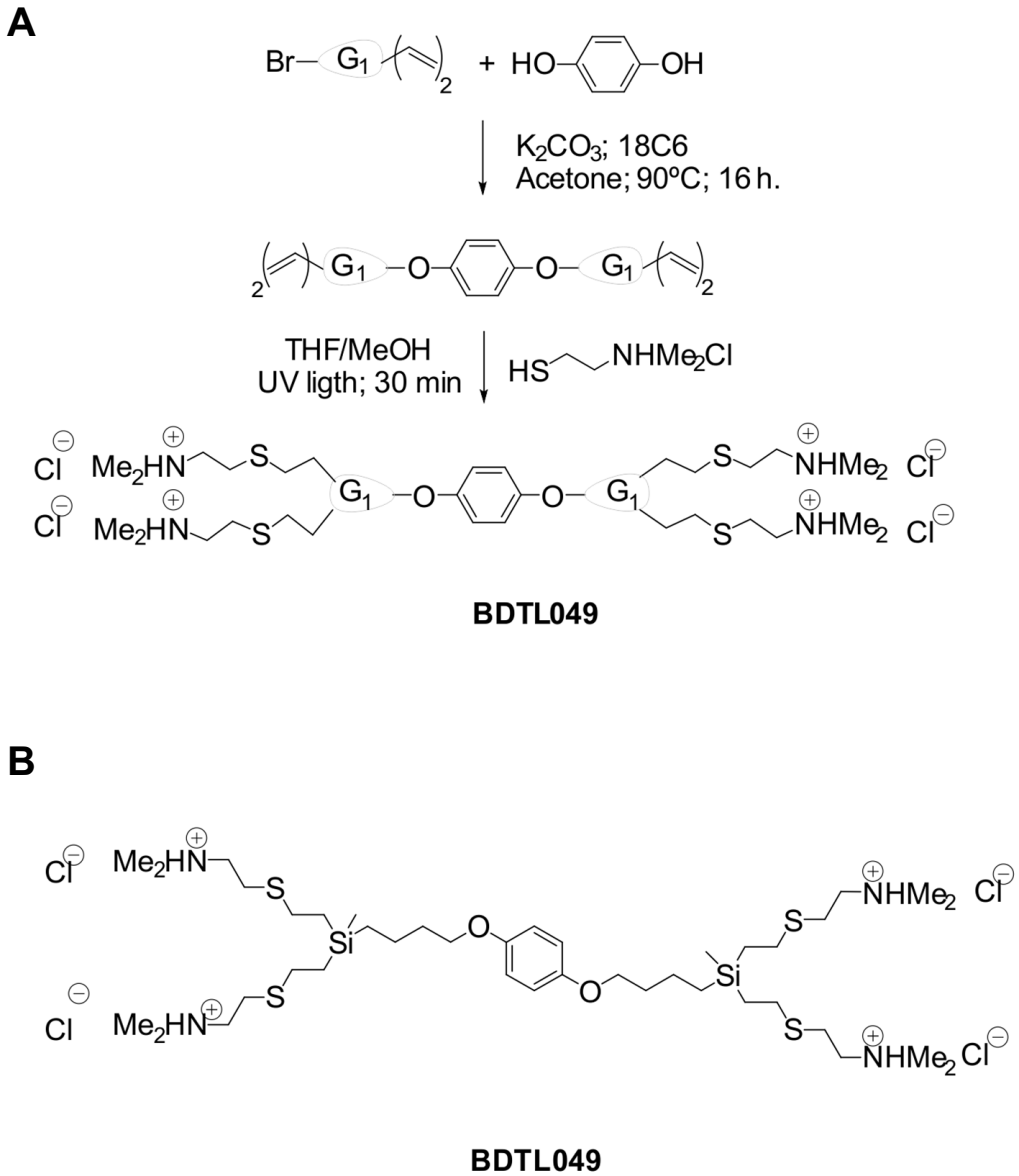
Synthesis, structure, and properties of the cationic carbosilane derivative used in this study. Synthetic procedure for the formation of compound BDTL49 **(A)** and its corresponding chemical structure **(B)**. G1 denotes first generation of the carbosilane scaffold. Molecular weight of 964 Daltons. The compound is air and water stable, soluble in protic solvents like water or methanol, and non-soluble in organic solvents.

**Figure 2:**
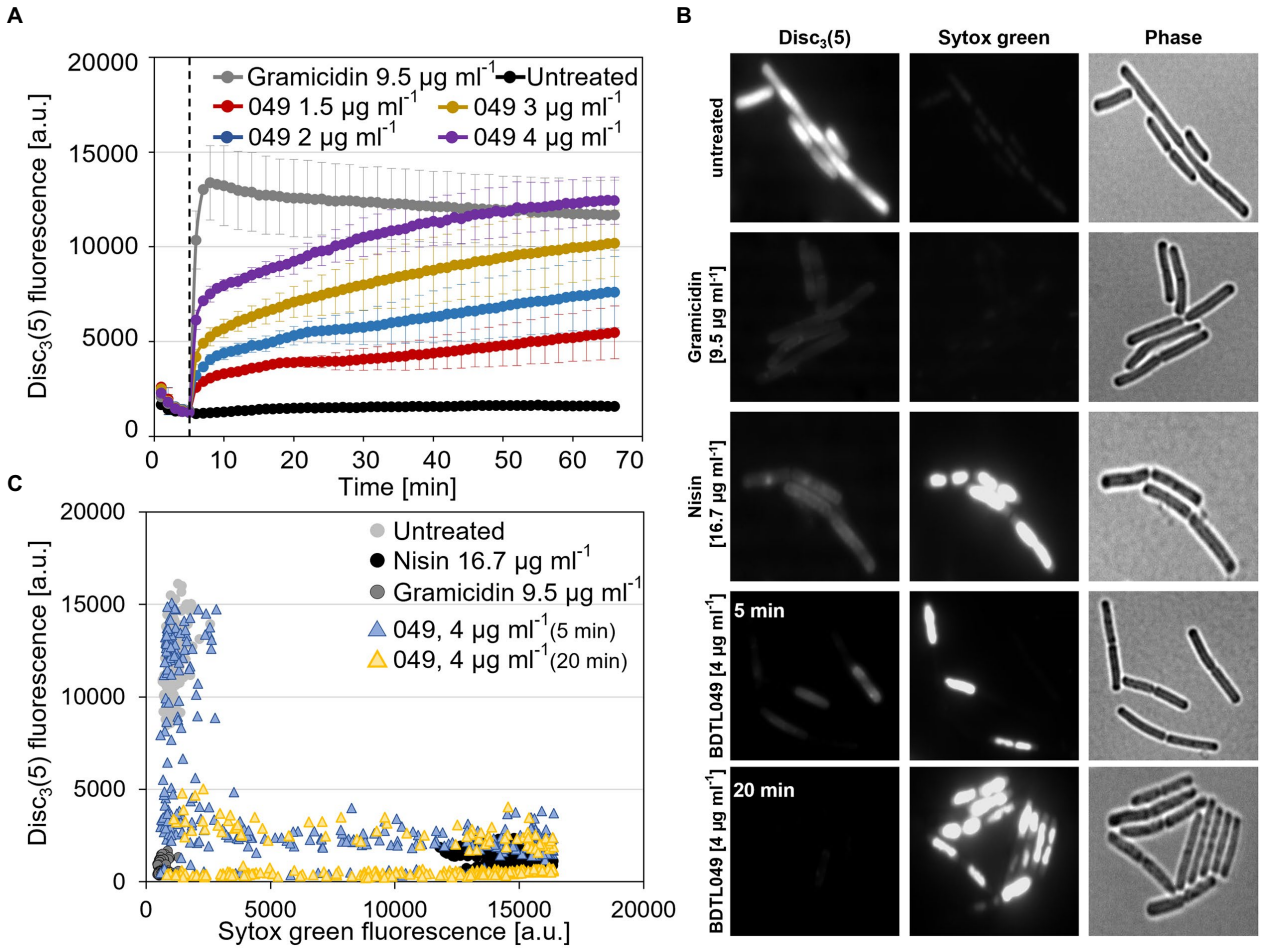
RNA-seq results upon BDTL049 treatment. **(A)** Volcano plot of differential expressed *Bacillus subtilis* genes. **(B)** Genes in **(A)** were clustered according to their Subtiwiki (Zhu and Stulke, 2018) classification. Upregulated genes are marked in light gray whereas downregulated genes are highlighted in black. For applied cut-off values regarding significance see the section “Materials and methods.”

**Figure 3:**
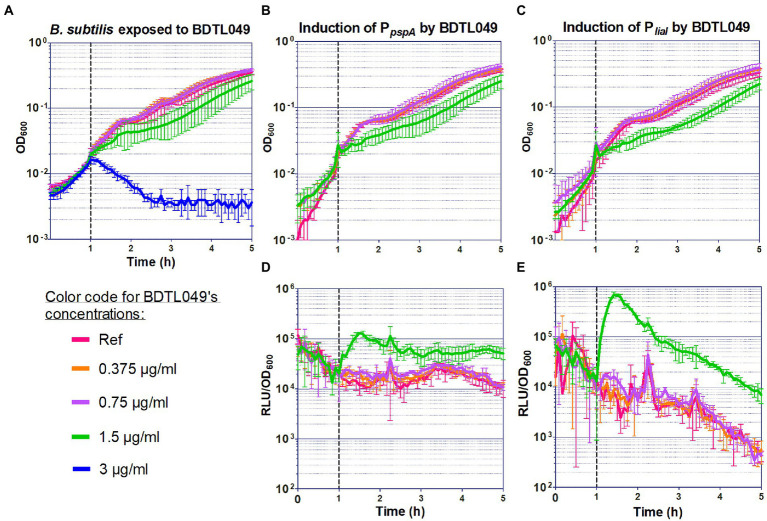
Sensitivity of *Bacillus subtilis* toward BDTL049, and induction of whole cell biosensors by BDTL049. **(A)** The effect of the addition of increasing concentrations of BDTL049 on the growth of exponentially growing wild type cells (*B. subtilis* W168) was determined by monitoring OD_600_ over time. The compound concentrations are indicated below the graph. **(B–E)** Induction of the P*_pspA_* **(B,D)** and P*_liaI_* **(C,E)** promoters by BDTL049 in liquid media. The effect of antibiotic exposure on growth is indicated as OD_600_ **(B,C)**, and promoter induction as relative luminescence units by OD_600_ **(D,E)**. The time of antibiotic addition is indicated by a vertical dashed line. The antibiotic concentrations used are color-coded according to graph **(A)**. The results presented in **(B–E)** correspond to strains TMB2299 (P*_pspA_*) and TMB3822 (P*_liaI_*). All experiments were performed at least in triplicate. Means and SDs are depicted.

**Figure 4:**
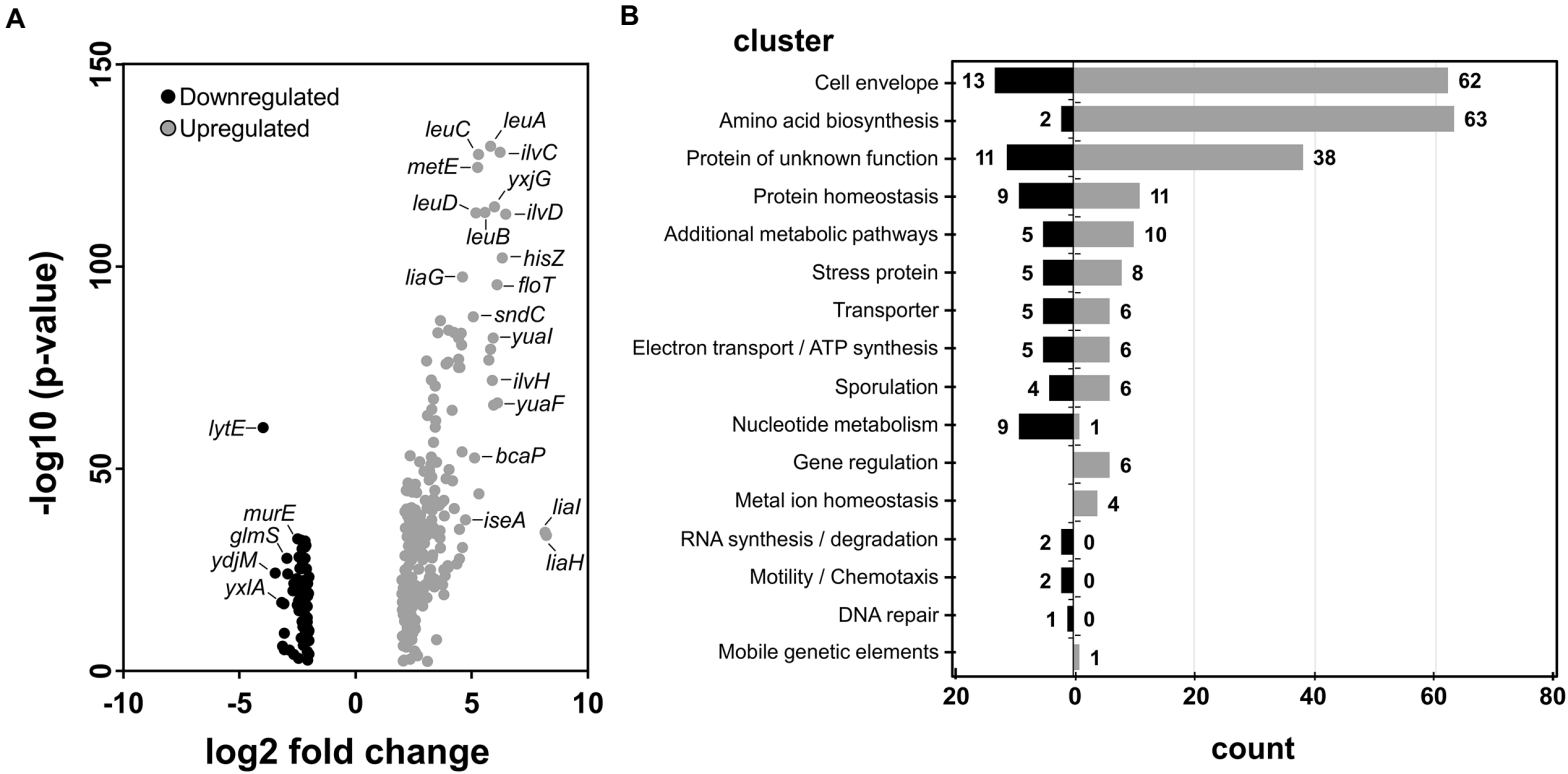
BDTL049 depolarizes the membrane and leads to pore formation. **(A)** Plate reader bulk measurement using the voltage-sensitive dye Disc_3_(5), showing membrane depolarization effects of wild type *Bacillus subtilis* cells upon treatment with different concentrations of the SynAnt BDTL049. The time point of compound addition is indicated by a dashed line. The peptide antibiotic Gramicidin as well as untreated cells are depicted and serve as controls. **(B)** Exemplary phase contrast (right panels) and fluorescence microscopy pictures of wild type *B. subtilis* cells stained with the voltage-sensitive dye Disc_3_(5) (left panels) and the DNA binding dye Sytox Green (middle panels) in the absence or presence of 4 μg ml^−1^ BDTL049. Incubation of cells with compounds was set to 5 min or as stated otherwise. As positive controls, cells were treated with 9.5 μg ml^−1^(equivalent to 5 μM) Gramicidin (depolarization without pore formation) or 16.7 μg ml^−1^ (equivalent to 5 μM) Nisin (depolarization through pore formation). **(C)** Scatter plot showing fluorescence intensities of Disc_3_(5) and Sytox Green derived from individual cells (*n* > 200). The data shown in graphs **(A,C)** derive from at least three independent biological replicates.

The present work followed such a *de novo* synthetic approach for the creation of a novel antimicrobial compound. BDTL049 is a rationally designed bow-tie topology dendrimer based on a carbosilane scaffold (see Figure 1). The antimicrobial activity of BDTL049 was comprehensively assessed *in vivo,* and its mode of action was investigated. For that, we used the Gram-positive model bacterium *Bacillus subtilis*. Our results demonstrate that BDTL049 acts in the concentration range of μg ml^−1^, comparable to other clinically established and frequently applied natural antibiotics (Table 1). BDTL049 dramatically impacts the fitness of *B. subtilis* by inducing, among others, a strong cell envelope stress response (Figure 2 and Table 2). Based on the combined results of whole-cell biosensors (Figure 3) and voltage-sensitive dyes (Figure 4), we identified the cytoplasmic membrane as the primary cellular target of this new antimicrobial compound. BDTL049 causes a collapse of the membrane potential by pore-formation and kills *B. subtilis* cells within minutes. Our results show that the bow-tie topology and chemical composition of the dendritic system BDTL049 constitute a promising lead structure for the development of novel antimicrobial agents, and that understanding its mechanism of action can pave the way for the rational design of yet more potent antibiotics.

**Table 1:** Minimal inhibitory concentration (MIC) and minimal bactericidal concentrations (MBC) of depicted antibiotics against *B. subtilis* W168.

	**Nisin**	**Gramicidin**_**ABCD**_	**BDTL049**	**Chloramphenicol**	**Kanamycin**	**Trimethoprim**	**Rifampicin**
**MIC**_**8h**_	6.25	4	4	2–4	1	0.5	0.0625
**MBC**	6.25	8	4–8	32	0.5	0.5	0.125–0.25

MIC values are shown at 8 h timepoints and MBC was determined after 24 h (see Materials and methods). All concentrations are shown in μg ml^−1^.

**Table 2:** Selection of the RNA-seq profile of differential expressed genes in *B. subtilis* upon BDTL049 exposure.

**Gene(s)**	**log2 fold change** ^**1**^	***p*-value** ^**1**^	**Regulation** ^**2**^	**Function** ^**2**^	**Cluster** ^**2**^
*liaIH*	8.22	2.8·10^−34^	LiaR	Protection against envelope stress	Cell envelope
*ilvD*	6.47	1.1·10^−113^	CodY	Biosynthesis of branched-chain amino acids	Amino acid/ nitrogen metabolism
*hisZGDBHAFI*	6.31	8.4·10^−103^	YlxR	Biosynthesis of histidine	Amino acid/ nitrogen metabolism
*ilvBHC-leuABCD*	6.23	6.2·10^−129^	TnrA, CodY, CcpA	Biosynthesis of branched-chain amino acids	Amino acid/ nitrogen metabolism
*yuaFI-floT*	6.13	4.9·10^−67^	SigW	Control of membrane fluidity	Cell envelope
*yxjG*	5.98	1.6·10^−115^	N/A	Putative methionine synthase	Amino acid/ nitrogen metabolism
*metE*	5.26	2.9·10^−125^	N/A	Biosynthesis of methionine	Amino acid/ nitrogen metabolism
*bcaP*	5.12	1.8·10^−53^	CodY	Biosynthesis/acquisition of branched-chain amino acids	Amino acid/ nitrogen metabolism
*sndC*	5.08	3.0·10^−88^	N/A	Sulfur metabolism / cysteine breakdown	Protein of unknown function
*iseA*	4.74	4.1·10^−38^	WalR	Protection against envelope stress	Cell envelope
*ypzA*	−2.85	7.2·10^−6^	SigG	Unknown	Sporulation
*cwlO*	−2.91	1.0·10^−24^	WalR	Cell wall synthesis, cell elongation	Cell envelope
*glmS*	−2.95	1.2·10^−28^	SigA	Cell wall synthesis	Cell envelope
*pbuG*	−3.06	5.5·10^−6^	PurR	Hypoxanthine and guanine uptake	Transporter
*xpt-pbuX*	−3.06	4.5·10^−10^	PurR	Purine salvage and interconversion	Nucleotide metabolism
*nupG*	−3.09	2.5·10^−17^	N/A	Purine uptake	Transporter
*pyrR*	−3.15	6.5·10^−7^	PyrR	Regulation of pyrimidine biosynthesis	Nucleotide metabolism
*yxlA*	−3.17	9.9·10^−18^	N/A	Putative purine-cytosine permease	Transporter
*ydjMN*	−3.45	5.3·10^−25^	WalR, PhoP	May be involved in cell wall metabolism	Cell envelope
*lytE*	−3.98	7.0·10^−61^	WalR, Spo0A, SigH, and SigI	Major autolysin, cell elongation, and separation	Cell envelope

Top 10 up- and downregulated genes are shown. For full dataset, see Supplementary Table 1.

1 Depicted values are representative of the highest differential expressed gene in cases of operons.

2 According to Subtiwiki (Zhu and Stulke, 2018).

## Materials and methods

### Chemical synthesis of BDTL049

The dendritic compound BDTL049 used in this work was synthetized following a similar protocol described previously (Lozano-Cruz et al., 2020). Briefly, a mixture of dendron BrG_2_V_2_ (0.79 g, 3.39 mmol), hydroquinone (0.19 g, 1.70 mmol), K_2_CO_3_ (0.92 g, 6.70 mmol), and crown ether (0.082 equiv.) in acetone was stirred at 90°C for 24 h. Then, the solution was filtered, and the solvent evaporated at reduced pressure. The oil obtained was extracted into hexane, the organic phase dried with MgSO_4_, and SiO_2_ added to remove the crown ether. The solution was filtered, and the solvent removed under vacuum. The residue was purified by using a size-exclusion chromatographic column, obtaining the final compound as yellow oil (0.43 g, 61%). After, the isolated vinyl dendrimer (0.50 g, 1.20 mmol), 2-(N, N-dimethylamino) ethanethiol hydrochloride (0.68 g, 4.84 mmol), 5 mol% 2,2-dimethoxy-2-phenylacetophenone (DMPA; 0.25 g, 1.0 mmol), and THF/methanol (1:2, 10 ml) were combined. The reaction mixture was deoxygenated and irradiated for 1.5 h. Another 5 mol% DMPA was then added, and the reaction mixture irradiated for a further 1.5 h, while monitored by 1H NMR spectroscopy. The initial reaction mixture was concentrated by rotary evaporation and redissolved in water. Afterward, the purification was performed using a size-exclusion chromatographic column, giving the final compound as a yellow solid (0.92 g, 80%). See Figure 1.

### Bacterial strains, plasmids and growth conditions

Table 3 lists the strains used in this study. *Escherichia coli* DH10β was used as an intermediate host for cloning purposes. *Bacillus subtilis* and *E. coli* cells were routinely grown in LB-Broth (Luria/Miller) for molecular biology (tryptone 10 g l^−1^, yeast extract 5 g l^−1^, NaCl 10 g l^−1^, and pH 7.0; Carl Roth, Karlsruhe, Germany) at 37°C with agitation. 1.5% (w/v) agar (Agar-Agar Kobe I, Carl Roth, Karlsruhe, Germany) was added to prepare the corresponding solid media. Strains were stored at −80°C in their corresponding growth media containing 20% (v/v) glycerol (Carl Roth, Karlsruhe, Germany). Ampicillin (Carl Roth, Karlsruhe, Germany) 100 μg ml^−1^ was used for selection of *E. coli*, when required. Chloramphenicol (Sigma-Aldrich, Merck KGaA, Darmstadt, Germany), 5 μg ml^−1^ was added to select *B. subtilis* biosensors, where appropriate.

**Table 3:** Bacterial strains used in this study.

Strain	Description^a^	Source /reference
*E. coli* DH10β	F-mcrA Δ(*mrr*-*hsdRMS*-*mcrBC*) Φ80d*lacZ*Δ*M15* Δ*lacX74 endA1 recA1 deoR* Δ(*ara,leu*)7697 *araD139 galU galK nupG rpsL* λ^−^	Lab collection
*E. coli CECT 515*	*Escherichia coli* (Migula 1895) Castellani and Chalmers 1919. Serotype O1:K1(L1):H7. NCTC 9001.	Spanish Type Culture Collection (CECT)
*S. aureus CECT 240*	*Staphylococcus aureus* subsp. *aureus* Rosenbach 1884. ATCC 6538P.	Spanish Type Culture Collection (CECT)
*B. subtilis*		
W168	Wild type; *trpC2*	Lab collection
*Reporter strains*		
TMB1620	W168 *sacA*:: *cm^r^* pCH*lux*104 (P*_bcrC_*-*lux*)	Höfler et al., 2016
TMB2009	W168 *sacA*:: *cm^r^* pJH*lux*104 (P*_psdA_*-*lux*)	Lab collection
TMB2299	W168 *sacA*:: *cm^r^* pASp3C*lux*01 (P*_pspA_*-*lux*)	Lab collection
TMB3417	W168 *sacA*:: *cm^r^* pM133C*lux*03 (P*_bceA_*-*lux*)	Lab collection
TMB3441	W168 *sacA*:: *cm^r^* pM133C*lux*06 (P*_lepA_-lux*)	Lab collection
TMB3561	W168 *sacA*:: *cm^r^* pSK3C*lux*01 (P*_yxdL_-lux*)	Lab collection
TMB3762	W168 *sacA*:: *cm^r^* pBS3C-P*_dinB_-lux*	This study; Hutter et al., 2004
TMB3763	W168 *sacA*:: *cm^r^* pBS3C-P*_yorB_-lux*	This study; Hutter et al., 2004
TMB3764	W168 *sacA*:: *cm^r^* pBS3C-P*_yvgS_-lux*	This study; Hutter et al., 2004
TMB3791	W168 *sacA*:: *cm^r^* pBS3C-P*_fabHB_-lux*	This study; Hutter et al., 2004
TMB3822	W168 *sacA*:: *cm^r^* pBS3C*lux*-P*_liaI_*	Popp et al., 2017
TMB4071	W168 *sacA*:: *cm^r^* pBS3C-P*_bmrBCD_-lux*	This study; Urban et al., 2007
TMB5600	W168 ∆*lnrLMN sacA::cm^r^* P*_lnrL231_-luxABCDE*	Revilla-Guarinos et al., 2020

a *cm*^r^, chloramphenicol resistance.

### Construction of transcriptional promoter-*luxABCDE* fusions

All vectors and plasmids used in this study are listed in Table 4; all oligonucleotides used in this study are listed in Table 5. Ectopic integrations within the *B. subtilis sacA* gene of the different promoter-*luxABCDE* fusion fragments were constructed based on the vector pBs3C-*lux* (Radeck et al., 2013). The promoter fragments were generated by PCR from genomic DNA with specific primers (Table 5) covering whole intergenic regions up to the potential ribosome binding site. After transformation into *E. coli* DH10β, ampicillin resistant colonies were checked by PCR with primers TM2262/2263, the inserts were verified by DNA sequencing and the resulting correct pBs3C-derived plasmids (Table 4) were linearized with *ScaI* and used to transform *B. subtilis*. Correct integration into the *sacA* locus was verified by amplification of an *up-* and *down-PCR* fragment with primers TM2505/2506 and TM5955/5956 or TM2507/2508, respectively. In each case, two independent positive clones were selected as reporter strains.

**Table 4:** Vector and plasmids used in this study.

Name	Description (primers used for cloning/antibiotic resistances)^a^	Source or reference
Vector		
pBs3C*lux*	pAH328 derivative; *amp*^r^, *cm*^r^, *sacA*’…‘*sacA*, *luxABCDE*	Radeck et al., 2013
Plasmids		
pBs3C-P_*dinB*_*-lux*	TM5078/TM5079; *cm^r^, amp^r^*	This study
pBs3C-P_*yvgS*_*-lux*	TM5080/TM5081; *cm^r^, amp^r^*	This study
pBs3C-P_*fabHB*_*-lux*	TM5082/TM5083; *cm^r^, amp^r^*	This study
pBs3C-P*_yorB_-lux*	TM5076/TM5077; *cm^r^, amp^r^*	This study
pBs3C-P*_bmrBCD_-lux*	TM5086/TM5087; *cm^r^, amp^r^*	This study

a *cm*^r^, chloramphenicol resistance, and *amp*^r^, ampicillin resistance.

**Table 5:** Oligonucleotides used in this study.

Name and purpose		Description (sequence)^a^	Use
Promoter fusions			
TM5076	PyorB-XbaI.fw	AAAAtctagaACGGAGGTCTATATTGTGAG	Whole-cell biosensors
TM5077	PyorB-PstI.rv	AAAActgcagGTTTTGAAATTTTTGGTACTAC	
TM5078	PdinB-XbaI.fw	AAAAtctagaGTGTTCCTCATCTATATCATCAATC	
TM5079	PdinB-PstI.rv	AAAActgcagCGTGTGTATAGCTTTCATTATAC	
TM5080	PyvgS-XbaI.fw	AAAAtctagaTGCTGAAGCATTGGAATAAGTG	
TM5081	PyvgS-PstI.rv	AAAActgcagAAATAGTTGACAAACATAGATGAAATAC	
TM5082	PfabHB-XbaI.fw	AAAAtctagaATCGCATCATCAAATACCTTCC	
TM5083	PfabHB-PstI.rv	AAAActgcagATGGTCAGATTATAACACTAGATATTAG	
TM5086	Pbmr-XbaI.fw	AAAAtctagaCGATGACGGTCTGATTGTCTTTC	
TM5087	Pbmr-PstI.rv	AAAActgcagATCAGCCGCCTTCTATTTTTTCCTTG	
Check primers for pBs3C*lux*			
TM2262	pAH328checkfwd	GAGCGTAGCGAAAAATCC	sequencing
TM2263	pAH328checkrev	GAAATGATGCTCCAGTAACC	
TM2505	pAH328 sacA front check fwd	CTGATTGGCATGGCGATTGC	integration of up fragment into genome
TM2506	pAH328 sacA front check rev	ACAGCTCCAGATCCTCTACG	
TM5955	pBS3Clux back check rev	GCAGCCTTTTCCAAACATTCCG	Integration of down fragment into genome
TM5956	pBS3Clux back check fwd	GATAGTTGATATCCAGCAGGATC	
TM2507	pAH328 sacA back check fwd	GTCGCTACCATTACCAGTTG	
TM2508	pAH328 sacA back check rev	TCCAAACATTCCGGTGTTATC	

a Sequences are given in the 5′ → 3’direction. Restriction sites are shown in lowercase.

### Determination of minimal inhibitory concentration and minimal bactericidal concentration

The sensitivity experiments with *B. subtilis* were performed in Mueller-Hinton (MH) Broth (Carl Roth, Karlsruhe, Germany), with three biological replicas and technical replicas in different days (Supplementary Figure 1). The overnight cultures (3 ml) were prepared by picking single colonies from fresh plates. The day cultures were inoculated from freshly grown overnight cultures at an OD_600_ of 0.05 in fresh medium containing 2-fold serial dilutions of the compound under study and plated into 96-well microtiter plates (100 μl per well). The plates were incubated at 37°C with constant middle shacking, in a Synergy™ NEOALPHAB multi-mode microplate reader (BioTek®, Winooski, VT, United States). Growth was monitored for 19 h by changes in OD_600_. The Minimal Inhibitory Concentration (MIC) was defined as the lowest concentration of antimicrobial agent needed to completely inhibit the bacterial growth at 8 h (MIC_8h_) to compare the effect of different antibiotics, which may be degraded over time and lose activity (own experimental observation with some antimicrobial peptides), and at 19 h (MIC_19h_) to compare the effect of BDTL049 on different bacterial strains.

The sensitivity assays with *E. coli* (CECT 515) and *Staphylococcus aureus* [CECT 240; obtained from the Spanish Type Culture Collection (CECT)] were performed as previously reported (Fuentes-Paniagua et al., 2016). Briefly, the MIC assays were run in duplicate microplates and three different wells for each concentration analyzed in the microplate. Dilutions of BDTL049 were prepared in Mueller Hinton Broth (Scharlau, ref. 02–136) inoculated with 5 μl of a bacteria suspension of 2 × 10^7^ CFU ml^−1^. Microplates were incubated at 37°C for 19 h using an ultra-microplate reader ELX808iu (Bio-Tek Instruments), considering the MIC the minimal concentration for which no turbidity was observed.

To determine the Minimal Bactericidal Concentration (MBC), 3 μl of the cultures used for the MIC assessment were droplet-plated in MH-agar. The plates were incubated for 24 h at 37°C, and the MBC was defined as the lowest concentration were no colonies indicative of cell growth, were observed.

### Sensitivity and promoter induction assays with exponentially growing planktonic cultures

The experiments testing the sensitivity of W168 exponentially growing cultures to BDTL049 were performed in Mueller Hinton (MH) Broth for antibiotic-sensitivity testing [beef infusion 2 g l^−1^, casein peptone (acidic hydrolysate) 17.5 g l^−1^, corn starch 1.5 g l^−1^, and pH value 7.4 ± 0.2; Carl Roth, Karlsruhe, Germany], with at least three biological replicas in different days. The overnight cultures (3 ml) were prepared by picking single colonies from fresh plates, and the day cultures (10 ml) were inoculated 1:200 with the overnight cultures and incubated at 37°C (220 rpm) until an OD_600_ of around 0.2 was reached. Then, the cell suspensions were diluted to an OD_600_ of 0.01, they were distributed into a 96-well transparent plate (95 μl per well) and incubated at 37°C (continuous middle shacking) in the Synergy™ NeoalphaB plate reader (BioTek®, Winooski, VT, United States). After 1 h of incubation, 5 μl of the synthetic compounds (at 20 times the desired final concentrations) were added to the wells, with one well left untreated as control. The incubation at 37°C with continuous middle shaking was continued for further 18 h. OD_600_ was measured every 5 min to monitor the growth rate.

For the whole-cell biosensors induction assays, antibiotic selection was added to the overnight cultures, but the day cultures (10 ml) were inoculated 1:200 with the overnight cultures without antibiotic selection. The same procedure as for testing the sensitivity of exponentially growing cultures was followed to determine the induction of the promoter-*luxABCDE* transcriptional fusions but the cells were plated in black 96-well plates (black, clear bottom; Greiner Bio-One, Frickenhausen, Germany) and besides OD_600_, luminesce between 300 and 700 nm was monitored every 5 min for at least 18 h.

### RNA sample preparation and sequencing

RNA-seq experiments were performed in triplicates with *B. subtilis* WT (BaSysBio; Anagnostopoulos and Spizizen, 1961; Nicolas et al., 2012) in LB Broth (Miller; tryptone 10 g l^−1^, yeast extract 5 g l^−1^, NaCl 10 g l^−1^, and pH 6.8–7.2; Sigma L3522). Day cultures were inoculated from overnight cultures and grown until mid-exponential phase (OD_600_ approx. 0.4) at 37°C. Subsequently, a second day culture of 200 ml LB Broth (Miller; Sigma L3522) was started to an OD_600_ = 0.1. Once this second day culture reached OD_600_ = 0.5, cells were split into 25 ml aliquots and either exposed to 4 μg ml^−1^ BDTL049 (final concentration) or remained untreated for 10 min. This concentration of BDTL049 was below the MIC in LB (Supplementary Figure 2). After treatment, cells were transferred to 50 ml falcons and growth was immediately stopped in an ice water bath followed by centrifugation at 8,000 rpm at 4°C for 3 min. The supernatants were discarded, and the resulting pellets were stored at −80°C. RNA isolation was performed with a phenol-chloroform extraction method as previously described (Popp et al., 2020). The cDNA library was prepared using the NEB Ultra RNA directional prep kit for Illumina and sequencing was performed on an Illumina HiSeq3000 system. Sequencing reads were mapped to the BaSysBio 168 strain (NC_000964.3) using Bowtie2 (Langmead and Salzberg, 2012). The software program featureCounts of the Subread package (Liao et al., 2014) was applied to generate counts for known genes. Differentially expressed genes were identified using the R/Bioconductor package DESeq2 (Love et al., 2014). A cut-off threshold of ±2 log2 fold change with a value of *p* lower than 0.001 was applied. The raw and processed RNA sequencing data obtained in this study has been deposited at the NCBIs Gene Expression Omnibus (Edgar et al., 2002) and is accessible *via* the GEO accession number GSE149270.

### Fluorometric measurement of membrane depolarization

Membrane depolarization assays based on the voltage sensitive Disc_3_(5) dye (Anaspec) were performed to further investigate the mode of action of BDTL049. This assay is well described elsewhere (te Winkel et al., 2016). In brief, *B. subtilis* wild type cultures were grown in MH (Mueller Hinton) Broth for antibiotic-sensitivity testing (Carl Roth, Karlsruhe, Germany) overnight and refreshed 1:200 in a day culture. Once the cells reached exponential growth phase (OD_600_ = 0.2–0.8), cells were diluted to an OD_600_ = 0.2 in fresh media supplemented with BSA (0.5 mg ml^−1^). Diluted cells were then transferred into a 96-well plate (black, clear bottom; Greiner Bio-One, Frickenhausen, Germany), and the auto fluorescence (excitation 610 nm and emission 660 nm) was monitored for 3 min using the Synergy™ NeoalphaB plate reader (BioTek, Winooski, VT, United States). After that, Disc_3_(5) was added to a final concentration of 1 μM. The incorporation of the dye into the membrane was followed in the plate reader for another 7 min till steady fluorescence values were reached. The antimicrobials were then added to the final concentrations as indicated in Figure 4A. Finally, changes in fluorescence were monitored for an additional hour. As a control experiment to eliminate misinterpretation of depolarization (i.e., observed changes in fluorescence) due to interactions between Disc_3_(5) and BDTL049, the procedure was repeated in the absence of cells. At least three biological independent experiments were performed whose mean fluorescence and standard deviations are depicted in Figure 4A.

### Analysis of membrane depolarization and pore formation with fluorescence microscopy

Fluorescence microscopy using Disc_3_(5) combined with Sytox Green was performed to verify the depolarization of *B. subtilis* cells by BDTL049 and investigate the potential of the synthetic antibiotic as membrane perturbing agent. Cells were cultivated and handled as described above. Cells were then incubated with 1 μM Disc_3_(5) and 50 nM Sytox Green (Thermo) for 5 min in an Eppendorf Thermomix under shacking conditions (800 rpm) and perforated lids to allow full aeration. After that, compounds of interest were added to the desired final concentrations (gramicidin 9.5 μg/ml, nisin 16.7 μg/ml, and BDTL049 4 μg/ml), followed by an incubation time of 5 min, and an additional incubation time of 20 min for BDTL049. Subsequently, 2 μl were transferred to an agarose pad (1% Ultra-pure Agarose, Invitrogen) and fluorescence microscopy was carried out using an Axio Observer 7 inverse microscope (Carl Zeiss, Jena, Germany) equipped with standard Cye5 (Ex: 650/ EM: 673) and eGFP (Ex: 488/EM: 509) filter sets. Microscopy pictures were analyzed using the tool Fiji (Schindelin et al., 2012) together with the plug-in MicrobeJ (Ducret et al., 2016). For each condition tested, a minimum of 200 cells from independent experiments, were marked as regions of interest to obtain both fluorescence intensities.

### Data analysis and statistical procedures

Sensitivity and promoter induction assays with exponentially growing planktonic cultures were performed with at least three biological replicas in different days in the case of the wild-type strain, and in biological duplicates (two clones) and technical triplicates (in different days) in the case of the whole-cell biosensors (luminescence measurements). From the values obtained for each time point, mean values and SD (±) were calculated and plotted (Figure 3). The sensitivity experiments with *B. subtilis* to determine the MIC were performed with biological and technical triplicates in different days. From the values obtained for each time point, mean values and SD (±) were calculated and plotted (Supplementary Figure 1A). MIC was calculated as presented in Supplementary Figure 1B. The sensitivity assays with *E. coli* (CECT 515) and *S. aureus* were run in duplicate microplates and three different wells for each concentration analyzed in the microplate considering the MIC the minimal concentration for which no turbidity was observed. The MBC was determined at least in triplicate with the cultures used for the MICs as presented in Supplementary Figure 1C. The data derived from the RNA-seq experiments were obtained in biological and technical triplicates. A cut-off threshold for gene expression differences of ±2 log2 fold change with a value of *p* lower than 0.001 was applied (Figure 2; Table 2). For the fluorometric measurement of membrane depolarization assays performed in the plate reader, each condition (antimicrobial compound) was tested in at least three biological independent experiments. From the raw data, mean values and SD (±) were calculated and plotted as function of time (Figure 4A). Depolarization and Sytox Green assays performed microscopically were evaluated using Fiji and the plugin MicrobeJ (Schindelin et al., 2012; Ducret et al., 2016). Here, pictures obtained from biological and technical triplicates were analyzed and regions of interest (i.e., cells) were chosen by the algorithm and manually corrected if necessary. Finally, from each condition tested, at least 200 cells from independent experiments were included in the final dataset and mean pixel intensities were plotted (Figures 4B,C).

## Results and discussion

### Synthesis and characterization of BDTL049, a carbosilane dendrimer with bow-tie type topology

Multiple aspects of natural antibiotics and other previously designed synthetic antibiotics were incorporated to generate the novel carbosilane bow-tie dendrimer BDTL049. The compound was synthetized in two simple steps (see Figure 1A and the section “Materials and methods”) as described previously (Lozano-Cruz et al., 2020). The structure of the bow-tie dendrimer BDTL049 is shown in Figure 1B. The antibiotic type and its final design was selected based mainly on data obtained previously for cationic ammonium-or imidazolium-terminated carbosilane dendritic systems of different topologies (Rodríguez-Prieto et al., 2020), for which the first generation with bow-tie topology has shown high antibacterial activity. In these systems, the antibacterial activity seems to be a compromise between the hydrophobicity established by the carbosilane structure and the hydrophilicity given by the peripheral cationic groups, along with topology (Fuentes-Paniagua et al., 2016).

### BDTL049 has antimicrobial activity comparable to natural antibiotics

We first determined the MIC and the MBC for BDTL049 against the Gram-positive model organism *B. subtilis* (Supplementary Figure 1) and compared the obtained values with a selection of antibiotics covering a range of different cellular targets (Table 1). BDTL049 treatment resulted in a MIC_8h_ value of 4 μg ml^−1^, which was comparable to the translational inhibitor chloramphenicol (2–4 μg ml^−1^) and the ionophore Gramicidin ABCD (4 μg ml^−1^). In contrast, *B. subtilis* was more susceptible toward the second ribosomal inhibitor, kanamycin (MIC_8h_ of 1 μg ml^−1^), the folic acid synthesis restraining compound Trimethoprim (MIC_8h_ of 0.5 μg ml^−1^), and particularly toward the RNA-polymerase interfering antibiotic Rifampicin, which yielded an almost 100-fold lower MIC_8h_ (0.0625 μg ml^−1^) compared to BDTL049.

The MBC results, reassembling the bactericidal concentration of the tested antimicrobials, showed that BDTL049 was as lethal for *B. subtilis* as Gramicidin ABCD or Nisin, with concentrations ranging between 4 and 8 μg ml^−1^, respectively. The MBC for Kanamycin, Trimethoprim, and Rifampicin was approximately 8–16-fold lower, while that of chloramphenicol was 4-fold higher than observed for BDTL049 (Table 1).

The antibacterial selectivity of BDTL049 with respect to the type of target bacteria was also investigated. MIC_19h_ and MBC values were determined for a second Gram-positive organism, *Staphylococcus aureus* CECT 240, and for the Gram-negative *Escherichia coli* CECT 515, and compared with the values of BDTL049 against *B. subtilis* W168. Both Gram-positives resulted equally sensitive to BDTL049 with MIC_19h_ values of 4 μg ml^−1^, and an MBC against *S. aureus* of 4 μg ml^−1^ and between 4 and 8 μg ml^−1^ for *B. subtilis* W168 (Supplementary Figure 1). Interestingly, the new compound behaved slightly better against *E. coli* with an MIC_19h_ value of 2 μg ml^−1^, and an MBC value also of 2 μg ml^−1^.

These results indicate that the new compound BDTL049 has antibacterial activity against Gram-positive and Gram-negative bacteria, and that it is as potent against *B. subtilis* as other classical antibiotics. Thus, we next aimed at identifying the mode of action (MOA) of this novel synthetic compound and its antimicrobial activity against the model organism *B. subtilis*.

### BDTL049 triggers a global transcriptional stress response in *Bacillus subtilis*

Numerous studies have established that the genome-wide gene expression signature of cells grown in the presence of sublethal concentrations of antimicrobial compounds provide a powerful indicator to narrow down the MOA of an antimicrobial compound (Wecke and Mascher, 2011; Popp et al., 2020). We therefore performed RNA-seq experiments aiming at unraveling the transcriptional response of exponentially growing *B. subtilis* cells when treated with 4 μg ml^−1^ BDTL049. Of 295 genes that were differentially expressed 10 min post BDTL049 addition (for details, see the section “Materials and methods”), 222 were upregulated, while 73 were inhibited relative to the non-treated control sample (Figure 2; Table 2).

Based on functional categories, the two most prominent cellular processes potentially targeted by BDTL049 are cell envelope integrity and amino acid metabolism (Figure 2B). But the latter has often been observed to strongly respond to cell wall antibiotic action for reasons still unknown (Wecke and Mascher, 2011). The same holds true for the genes involved in purine and pyrimidine salvage and biosynthesis pathways, which were repressed by BDTL049 (Table 2). This points toward the cell envelope as the primary target of BDTL049 action.

This assumption is supported by the strong BDTL049-dependent induction of the *liaIH* operon, which is controlled by the LiaRS TCS (Mascher et al., 2003, 2004; Jordan et al., 2006). The Lia system of *B. subtilis* specifically responds to cell envelope stress and the effector proteins LiaIH are thought to support envelope integrity at the site of damage (Wolf et al., 2010, 2012). This observation strongly suggests the cell envelope as critical target of BDTL049 action. Along these lines, the upregulation of the SigW-dependent *yuaF-floT-yuaI* operon potentially implies alterations of membrane fluidity state caused by BDTL049 action (Figure 2; Table 2; Bach and Bramkamp, 2013).

In contrast, the gene encoding the major autolytic endopeptidase of *B. subtilis*, *lytE*, was strongly downregulated upon BDTL049 treatment (Figure 2). LytE is known to interact with cell wall associated proteins at the division septum and during cell wall synthesis. Deletion of *lytE* increases resistance against beta-lactams, presumably by delaying cell lysis (Luo and Helmann, 2012). Thus, downregulation of *lytE* could be interpreted as a resistance mechanism against BDTL049 treatment. Similarly, the downregulation of the SigE-dependent *mur* operon, which is involved in peptidoglycan synthesis, points toward an indirect resistance mechanism. A recent study demonstrated that a point mutation in *ispA*, encoding a serine protease, together with repression of the *mur* operon enhances the formation of L-forms in *B. subtilis* populations (Kawai et al., 2018). L-forms cells, lacking a cell wall, are known to withstand cell wall antibiotics such as beta-lactams and antimicrobial peptides (Wolf et al., 2012; Kawai et al., 2018). In addition to the aforementioned targets, a number of BDTL049-inducible genes encode proteins of unknown function.

Taken together, we observed a distinct and strong transcriptional response of *B. subtilis* to BDTL049 treatment that points toward cell envelope homeostasis as the primary target, accompanied by disruption of other cellular processes. This prompted a more detailed analysis to mechanistically understand the action of BDTL049 as a potential antimicrobial agent.

### BDTL049 targets the bacterial cell envelope

The changes in gene expression occurring in *B. subtilis* after BDTL049 treatment pointed toward the cell envelope as the cellular target of this compound. We first verified this hypothesis by analyzing the response of a panel of *B. subtilis-*derived whole-cell biosensors specific for different cellular targets (Table 3), when exposed to BDTL049. Our collection included: six envelope stress-inducible promoters, P*_bcrC_*, P*_liaI_*, P*_pspA_*, P*_bceA_*, P*_psdA_*, and P*_yxdL_* (Mascher et al., 2004; Pietiäinen et al., 2005; Staroń et al., 2011; Müller et al., 2016; Radeck et al., 2016); the amphotericin-like polyenes biosensor P*_lnrL_* (Revilla-Guarinos et al., 2020); two DNA damage-inducible promoters, P*_dinB_* and P*_yorB_* (Cheo et al., 1991; Hutter et al., 2004; Urban et al., 2007); the transcription inhibitor-sensitive reporter strain P*_yvgS_* (Hutter et al., 2004; Urban et al., 2007); the fatty acids biosynthesis inhibitor reporter strain P*_fabHB_* (Hutter et al., 2004; Urban et al., 2007); the P*_bmrBCD_* reporter for translation inhibitors (Urban et al., 2007), as well as the constitutive promoter P*_lepA_* as a control (Radeck et al., 2013).

The response of these 13 whole-cell biosensors to a subinhibitory concentration of BDTL049 was investigated. This concentration was determined by a sensitivity assay, for which cells of *B. subtilis* W168 were challenged in early exponential phase with serial dilutions of BDTL049 (from 0.375 to 3 μg ml^−1^), assessing the effect on *B. subtilis* growth by OD_600_ determination (Figure 3A). While a concentration of 1.5 μg ml^−1^ impaired *B. subtilis* growth without completely inhibiting it, indicating that cellular damage was considerable but not lethal, the higher concentration tested (3 μg ml^−1^) completely abolished growth by inducing cell lysis (Figure 3A). A concentration of 1.5 μg ml^−1^ was therefore chosen for the biosensor assays, in which both the inhibitory effect of BDTL049 on bacterial growth (by OD_600_ measuring) and the induction of the promoters (based on luminescence readout) were monitored. Of the 13 reporter strains, only the promoters P*_liaI_* and P*_pspA_* were induced by BDTL049 (data not shown), and their activation occurs only upon reaching a damage-inducing threshold concentration of 1.5 μg ml^−1^ BDTL049, as seen by the effect on cellular growth (Figures 3B–E).

P*_liaI_* drives the expression of the *liaIH* operon, while P*_pspA_* controls *pspA*. Both LiaH and PspA belong to the PspA/IM30 phage shock protein family involved in bacterial envelope stress responses (Thurotte et al., 2017). The *liaIH* operon is upregulated in response to numerous antibiotics targeting membrane-anchored steps of cell wall biosynthesis, including bacitracin and lantibiotics (e.g., nisin, gallidermin, and mersacidin) and LiaH was proposed as a protein marker for interference with cell wall biosynthesis by lipid II binding (Wenzel et al., 2012). In contrast, expression of *pspA* is induced by nisin, gallidermin, gramicidin A, gramicidin S, and valinomycin, and PspA has been appointed as a protein marker for membrane integrity stress (Wenzel et al., 2012). The “off→on” induction of both promoters P*_liaI_* and P*_pspA_* upon reaching a damaging concentration of 1.5 μg ml^−1^ BDTL049 and the massive cellular death occurring at only slightly higher concentrations of 3 μg ml^−1^ (Figure 3A) might point toward pore formation in the cytoplasmic membrane when enough BDTL049 is present (upon reaching a threshold concentration), and LiaH and PspA binding and stabilizing the membrane in response to the damage.

### BDTL049 causes membrane depolarization and pore formation

Having identified the cell envelope—and presumably the membrane—as primary target of BDTL049 action, we next investigated the ability of this compound to interfere with membrane integrity. First, we analyzed if BDTL049 can cause membrane depolarization using the voltage-sensitive dye Disc_3_(5), which gets incorporated into well-energized membranes. The resulting accumulation of Disc_3_(5) on the membrane leads to quenching of the overall fluorescence in the cell culture. Compounds that alter the membrane energy state cause a release of Disc_3_(5) into the supernatant and thus subsequent dequenching, that can be followed fluorometrically (te Winkel et al., 2016).

Adding BDTL049 to Disc_3_(5) stained cells (dashed line, Figure 4A) resulted in a rapid increase of fluorescence, clearly indicating dissipation of membrane potential. This effect was pronounced for all concentrations, ranging from sub-lethal to MIC values, and followed a dose dependent manner. For all concentrations tested, the BDTL049-induced membrane depolarization was sustained over time and in any case the membrane potential was restored back to control levels. However, compared to the peptide antibiotic mix Gramicidin_ABCD_, a standard control for full membrane depolarization, similar fluorescence levels were only reached for the highest concentration of BDTL049 (4 μg ml^−1^) and only after approximately 40 min post induction.

We hypothesized, that the BDTL049-dependent depolarization could be subject of cell-to-cell heterogeneity, resulting in subsets of cells that differ in their degree of depolarization. To test this, we next performed fluorescence microscopy using Disc_3_(5) combined with Sytox Green, a membrane impermeable DNA stain used as reporter for pore formation (te Winkel et al., 2016; Kepplinger et al., 2018). Two peptide antibiotics, Nisin and Gramicidin_ABCD_, were included as positive controls for full homogenous membrane depolarization with and without pore formation, respectively (Kepplinger et al., 2018). As expected, untreated cells showed Disc_3_(5) fluorescence on intact membranes, without Sytox Green staining of the intracellular DNA (Figure 4B). Both control antibiotics (Gramicidin and Nisin) performed according to their expected mode of action after only 5 min of treatment (Figure 4B, rows 2 and 3). Addition of the small cation-specific channel-forming gramicidin (Kelkar and Chattopadhyay, 2007) led to membrane depolarization [loss of Disc_3_(5) fluorescence] without membrane permeabilization (no Sytox Green staining). The pore-forming Nisin (Wiedemann et al., 2004) led to both membrane depolarization [loss of Disc_3_(5) fluorescence] and membrane permeabilization, which allowed the passage of the otherwise membrane-impermeable DNA intercalating dye Sytox green resulting in strong fluorescent cells. Under the same experimental conditions (5 min incubation), BDTL049 treatment led to a strong cell-to-cell variance in membrane depolarization levels and only the subset of cells showing full membrane dissipation correlated with positive Sytox Green signals (Figure 4B, row 4). A homogeneous response of all cells was only observed after prolonging the incubation time of BDTL049 treatment to 20 min (Figure 4B, row 5).

We then quantified our microscopic observations by analyzing the Disc_3_(5) and Sytox green fluorescence intensities of at least 200 cells of each tested condition and plotted both fluorescence intensities (Figure 4C). Three well-isolated populations of cells were distinguishable for (i) the intact—untreated—control cells [high Disc3(5) and low Sytox Green fluorescence, pale-grey dots], (ii) the cells with depolarized but not permeabilized membranes after Gramicidin treatment [low Disc3(5) and low Sytox Green fluorescence, dark-grey dots], and (iii) the cells with depolarized and permeabilized membranes after Nisin treatment [low Disc3(5) and high Sytox Green fluorescence, black dots]. For BDTL049, the quantification again supported the previously observed heterogeneity in the MOA. After 5 min of treatment, two connected cell-populations transiting from one to the other could be identified (blue triangles in Figure 4C): one population with high Disc3(5) fluorescence and low Sytox Green fluorescence (indicative of intact membranes and no DNA staining), together with a second population with a marked loss of Disc3(5) fluorescence and an increasing Sytox green fluorescence (indicative of membrane depolarization accompanied with pore formation). Upon an increase of incubation time (yellow triangles in Figure 4C), we observed homogenous membrane depolarization of all cells [loss of Disc_3_(5) fluorescence] together with the progressive pore formation indicated by the DNA staining.

Taken together, our results demonstrate that BDTL049 exhibits a rapid initial depolarization of the cells that explains growth inhibitions even for sub-lethal concentrations (Figure 3A). However, due to the heterogeneous behavior of BDTL049 in pore formation, only the highest concentration (4 μg ml^−1^) with prolonged incubation time unleashed the full effectiveness of BDTL049 in killing *B. subtilis*.

## Conclusion and outlook

This study presents the design, synthesis, and comprehensive physiological characterization of a novel synthetic antibiotic, BDTL049, with antibacterial activity against Gram-positive and Gram-negative bacteria. This compound is a rationally designed synthetic ammonium-terminated carbosilane dendritic system, with a very hydrophobic inner skeleton and four net positive charges at its periphery that provide its hydrophilicity and therefore water-solubility. Moreover, this positive net charge is presumably crucial for interacting with the negatively charged cell envelope—a prerequisite to exhibit its antimicrobial action at the cytoplasmic membrane by mimicking the positive charge of many naturally occurring antimicrobial peptides. Once the cytoplasmic membrane is reached, BDTL049 seems to accumulate, which might also result on changes on the membrane fluidity as suggested by the upregulation of the *yuaF-floT-yuaI* operon (Figure 2; Table 2) involved in the control of membrane fluidity (Bach and Bramkamp, 2013). Additionally, BDTL049 forms pores in the membrane, which result in the loss of the membrane potential and in membrane permeabilization (Figure 4). Transcriptome analysis after exposure to BDTL049 highlighted the enormous stress exerted by this new synthetic antibiotic on *B. subtilis* as revealed by multiple changes in the cell surface homeostasis and central metabolism pathways (Figure 1; Table 1).

The damage-sensing response systems LiaIH and PspA are induced in an off–on manner when the cells are challenged with threatening concentrations of BDTL049 (Figure 3). This could indicate that a threshold concentration of BDTL049 must be reached for the synthetic antibiotic to accumulate on the cell membrane and induce enough pore formation as for it to be perceived as “active damage” by the cell surface stress response systems. The cell-to-cell variation in the degree of membrane penetration by BDLT049 (Figures 4B,C) probably accounts for the slow, dose-dependent depolarization curves (Figure 4A). This heterogeneity might suggest that differences in the cellular state of growth might result in different interactions with BDTL049. But the observation that longer incubation times resulted in consistent pore formation throughout the population, rather supports the hypothesis that a threshold-concentration of BDTL49 needs to accumulate on the membrane before sufficient damage can be caused to result in its killing activity. It should be noted that both hypotheses are not mutually exclusive, that is, the metabolic/growth state might as well influence the kinetics of BDTL049 accumulation at the (extracellular surface of) cytoplasmic membrane. The data generated in this work are consistent with a model that suggests that BDTL049 accumulates to form oligomeric structures that function as membrane pores. It remains elusive whether this requires a specific docking molecule on the cell membrane, which could serve as an anchoring point for BDTL049 oligomerization, comparably to nisin which at low concentrations binds to the cell-wall precursor lipid II, preventing cell wall biosynthesis, while using it as a anchoring molecule to initiate membrane insertion and pore formation when a higher threshold concentration of nisin is reached (Breukink et al., 1999; Wiedemann et al., 2001). Future studies will have to focus on testing this, so far, speculative ideas. But the data presented in this report are promising, since BDTL049 is active at concentrations within the same range of natural and clinically relevant antibiotics such as the pore former nisin and the ionophore gramicidin ABCD.

Future studies addressing the clinical application of the new compound will also have to focus on testing its cytotoxicity against animal cell lines. In our experience, toxicity of dendrimers depends on the generation (number of positives charges). First, generation compounds (four or six positives charges) afford lower toxicities compared with second generations (eight or 12 positive charges). The toxicity observed for dendrimers of first generation containing-NMe3 groups are around 150 μM for IC50 (Heredero-Bermejo et al., 2015; Fuentes-Paniagua et al., 2016). In the case of BDTL049 because of the presence of-NHMe2 groups we expect a slightly decrease of this number, but still higher enough compared to its antibacterial activity.

Our combined chemical and physiological results provide a promising entry point to apply our knowledge on natural antibiotic action for the rational design of new molecules with antimicrobial properties (Fjell et al., 2012; Torres et al., 2019). BDTL049 should now be used as a lead structure for modifying its chemical structure (and hence properties), in order to develop dendrimeric carbosilanes with bow-tie topologies into even better and stronger antimicrobials. These molecules are promising candidates to complement and expand the therapeutic power of natural antibiotics as result of an adequate hydrophilic-hydrophobic balance induced by the peripheral ammonium groups and the dendritic skeleton, respectively. These results open the door in searching for new candidates to fight antibiotic resistant pathogens.

## Data availability statement

The raw data supporting the conclusions of this article will be made available by the authors, without undue reservation, to any qualified researcher. The raw and processed RNA sequencing data obtained in this study has been deposited at the NCBIs Gene Expression Omnibus (Edgar et al., 2002) and is accessible via the GEO accession number GSE149270.

## Author contributions

AR-G, PP, FD, and TM conceived the study and discussed and planned the experiments. AR-G, PP, FD, and JH carried out the microbiological experiments. PP performed microscopy techniques. AR-G and PP performed data analysis. AR-G, PP, and TM wrote the manuscript. TL-C, RG, and FM designed the synthetic antibiotic BDTL049, which was chemically synthetized by TL-C under the supervision of RG and FM. FM and RG participated in discussion of the results. RG contributed to manuscript writing. All authors contributed to the article and approved the submitted version.

## Funding

This research was funded by SMWK Saxony (TG70 grant BACMOT 100315856 to AR-G) and by a grant from the Deutsche Forschungsgemeinschaft (MA2837/3 to TM) in the framework of the priority program SPP1617 “Phenotypic heterogeneity and sociobiology of bacterial populations.” FD thanks the TU Dresden Graduate Academy for a partial PhD scholarship. This research was also funded by grants from CTQ2017-86224-P and PID2020-112924RB-I00 (MINECO), consortium NANODENDMED II-CM (B2017/BMD-3703) and IMMUNOTHERCAN-CM (B2017/BMD3733) from Comunidad de Madrid. CIBER-BBN is an initiative funded by the VI National R&D&I Plan 2008–2011, Iniciativa Ingenio 2010, Consolider Program, and CIBER Actions and financed by the Instituto de Salud Carlos III with assistance from the European Regional Development Fund.

## Conflict of interest

The authors declare that the research was conducted in the absence of any commercial or financial relationships that could be construed as a potential conflict of interest.

## Publisher’s note

All claims expressed in this article are solely those of the authors and do not necessarily represent those of their affiliated organizations, or those of the publisher, the editors and the reviewers. Any product that may be evaluated in this article, or claim that may be made by its manufacturer, is not guaranteed or endorsed by the publisher.
