# Supplementary material for: Synthesis and mechanism-of-action of a novel synthetic antibiotic based on a dendritic system with bow-tie topology
Source: Front Microbiol. 2022 Aug 26;13:912536. doi: 10.3389/fmicb.2022.912536 (PMC9459136; doi:10.3389/fmicb.2022.912536)
Supplement: Supplementary file 2 [file Data_Sheet_1.docx]

Supplementary Material

# Supplementary Figures





**Supplementary Figure 1. Determination of MIC and MBC of BDTL049 for *B. subtilis* W168 in MH Broth.**

**A. Growth of W168 in the presence of serial dilutions of BDTL049 in MH Broth.** Cells were inoculated to an OD_600_ of 0.05 in MH with different concentrations of BDTL049. Cultures were incubated in aerobic conditions at 37°C. The time points for the MIC determinations presented in Graph B are indicated with vertical black dashed lines. Results present the mean and standard deviation of three replicas.

**B. MIC after 8 hours of growth and after 19 hours of growth in the presence of BDTL049.** The values of OD_600_ of W168 in the presence of serial dilutions of the antibiotic at the selected time points indicated in Graph A are presented as a function of the antibiotic concentration.

**C. Determination of the MBC of BDTL049 in MH.** 3 µl of the cultures used for the MIC assessment were droplet-plated in MH-agar. The plates were incubated for 24 hours at 37°C and the MBC was defined as the lowest concentration were no colonies indicative of cell growth, were observed.

In A and B, the results are the mean and standard deviation of three replicas. Five replicas are presented in C.


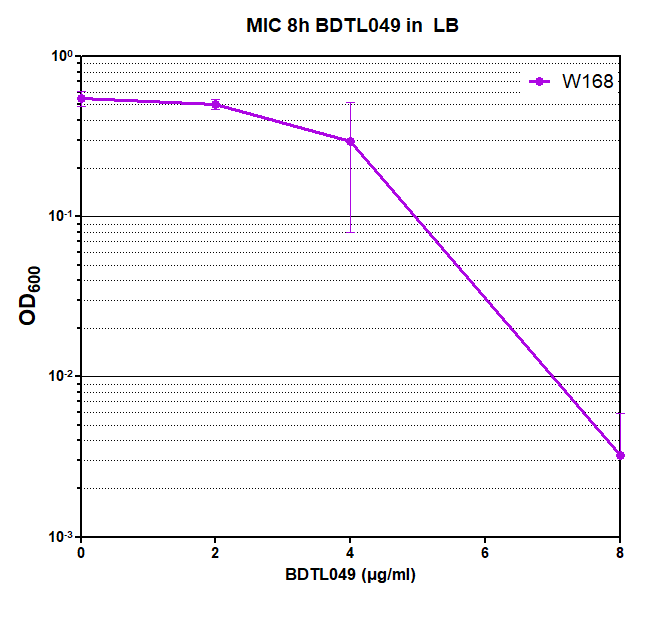


**Supplementary Figure 2.** **MIC_8h_ of BDTL049 for *B. subtilis* W168 in LB medium.** OD_600_ after 8 hours of growth in the presence of serial dilutions of the antibiotic is presented. Cells were inoculated to an OD_600_ of 0.05 in LB with different concentrations of BDTL049. Cultures were incubated in aerobic conditions at 37°C. MIC was defined as the lowest antibiotic concentration that completely inhibited growth at 8h. Mean and standard deviations of 4 replicas are presented.
